# Merged consensus clustering to assess and improve class discovery with microarray data

**DOI:** 10.1186/1471-2105-11-590

**Published:** 2010-12-03

**Authors:** T Ian Simpson, J Douglas Armstrong, Andrew P Jarman

**Affiliations:** 1 Genes and Development Group, Centre for Integrative Physiology, University of Edinburgh, Hugh Robson Building, George Square, Edinburgh, EH8 9XD, UK

## Abstract

**Background:**

One of the most commonly performed tasks when analysing high throughput gene expression data is to use clustering methods to classify the data into groups. There are a large number of methods available to perform clustering, but it is often unclear which method is best suited to the data and how to quantify the quality of the classifications produced.

**Results:**

Here we describe an R package containing methods to analyse the consistency of clustering results from any number of different clustering methods using resampling statistics. These methods allow the identification of the the best supported clusters and additionally rank cluster members by their fidelity within the cluster. These metrics allow us to compare the performance of different clustering algorithms under different experimental conditions and to select those that produce the most reliable clustering structures. We show the application of this method to simulated data, canonical gene expression experiments and our own novel analysis of genes involved in the specification of the peripheral nervous system in the fruitfly, *Drosophila melanogaster*.

**Conclusions:**

Our package enables users to apply the merged consensus clustering methodology conveniently within the R programming environment, providing both analysis and graphical display functions for exploring clustering approaches. It extends the basic principle of consensus clustering by allowing the merging of results between different methods to provide an averaged clustering robustness. We show that this extension is useful in correcting for the tendency of clustering algorithms to treat outliers differently within datasets. The R package, *clusterCons*, is freely available at CRAN and sourceforge under the GNU public licence.

## Background

The need to classify observations into groups based on shared properties is common to the analysis of many types of quantitative and qualitative biological data. One of the most common applications of classification is the segregation of high throughput gene expression measurements into groups based on specific criteria (e.g. co-expression, profile-shape over a time course, partitioning between patient categories). This is usually achieved by the application of clustering techniques in which the distance between features (e.g. genes) are calculated from the numerical data (e.g. gene expression values) and used to partition the data into discrete groups. An extensive range of methods have been developed for clustering data (for reviews see [1-4]). The plethora of both methods and parameters under which clustering can be performed presents a significant problem in selecting a clustering approach that is well suited to the data type. In addition the data needs to be processed in a way that provides the best opportunity to isolate well-defined and meaningful clusters from the data. The importance of being able to assess both the method and the parameters in a systematic and comparable manner is significant medically (e.g. patient classification, drug efficacy testing and treatment), more generally in bioscience research (e.g. classifying gene and protein groupings, prioritising candidate lists, pathway and network topological analysis) and in many other fields (e.g. financial systems, network communications, demographics). When clustering we want to know how many clusters there are and how confident we are that the clusters and their members are those best supported by the data.

The clustering of biological data is most commonly performed in an unsupervised manner since the classes to which the features belong are not known in advance. Many studies have focused on high throughput gene expression data where the expression of tens of thousands of genes are measured simultaneously and compared across multiple conditions. Indeed there have been approximately 50,000 published microarray studies in the last decade [5]. In these cases the high-dimensionality, noise and small condition number of the data sets makes cluster identification problematic. To aid in this task a range of metrics are used to assess the results of clustering experiments, mainly based on calculations of cluster compactness (intra-cluster variation), cluster separation (inter-cluster variation) including external, internal and relative criteria [6] and validity indices [7,8]. These measurements generally assess how well defined and separated clusters are without considering their stability or the confidence with which members can be assigned to any one cluster. A range of resampling approaches have been developed to quantify clustering tendency, stability and validity both for entire sets of clusters and for members of clusters [9-20]. Of these methods, Monti et al. [14] is the only one to develop a generalised, model independent resampling based methodology to assess cluster stability. This method, *consensus clustering*, can be applied to any clustering approach that produces a deterministic classification output.

We now report the development of an extended implementation of *consensus clustering *based on the methodology of Monti et al. [14] in the widely used statistical programming language R [21]. This extension allows the comparison and visualisation of the results of clustering experiments using any number of different clustering algorithms and parameters within a unified framework. In addition it provides methods to merge results to improve the quality of classifications. The premise of merging clustering results is that confidence in the classification is increased if similar results are produced using two or more methodologically different clustering algorithms (or in fact by using the same algorithm under very different conditions). We demonstrate the use of this *merge consensus clustering *methodology in simulated gene expression cases, canonical gene expression data from the leukaemia data set of Golub et al. [22] and temporal gene expression profiles captured from the development of the peripheral nervous system (PNS) of the fruitfly *Drosophila melanogaster *(unpublished data). We show that *merge consensus clustering *improves the quality of clustering results and provide quantitative measures of cluster and cluster membership robustness. These measures can be used to select the best methods and parameters for clustering a data set and allow the user to make informed decisions about the validity and composition of the resulting clusters. The *clusterCons *package has been developed to work with the clustering methods provided by the R package *cluster*, including *agnes *(agglomerative hierarchical), *diana *(divisive hierarchical), *pam *(partitional) and *k-means*. This is achieved via simple wrappers, which can be extended by the user to provide access to other clustering methods in R itself or in external applications called via R. The *clusterCons *package is easy to use and allows the user to perform clustering, robustness quantification and visualisation in the R environment facilitating the simple integration of analyses and exploiting the statistical and visualisation power of R.

## Implementation

The process of *consensus clustering *begins by randomly selecting a proportion of rows from the data and then clustering the subset using the currently specified clustering algorithm and parameters. This sampling and clustering is repeated many times to test the effect of removing features on the clustering result. The clusters produced by each iteration are stored in connectivity and indicator matrices which are later used to calculate a consensus clustering result. Features that are commonly found in the same cluster are, by definition, reliable cluster members, whereas those whose co-clustering is less frequent or dependent on the presence of other features are less reliable. The consensus clustering result is used to calculate cluster and membership robustness. We extend the consensus clustering method of Monti et al. [14] to *merge consensus clustering *in which we perform *consensus clustering *with many different clustering algorithms and/or parameters. The resulting consensus matrices are then merged by weighted averaging to produce a merge consensus matrix. This matrix can be used as a distance matrix in subsequent clustering experiments and to re-calculate cluster and membership robustness. The advantage of the merge consensus matrix is that it mitigates for the different classification properties of clustering algorithms, with some being more susceptible to outliers or particular types of data structure.

### Calculating the consensus clustering result

**Figure 1:**
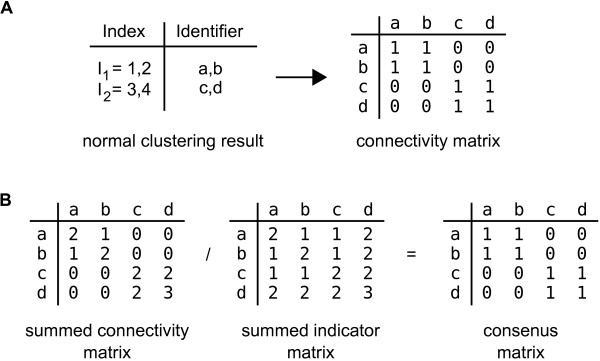
**Calculating the consensus clustering result**. The results of any discrete clustering algorithm can be represented as a membership list in which the features are indexed by cluster. (A) The clustering result can be readily converted into a connectivity matrix representing the co-clustering connections of the features. In a consensus clustering experiment the clustering process is performed many times with sub-samples of the data rows and the resulting partial connectivity matrices are summed. In addition, the frequency with which pairs of features are drawn together are counted and summed to produce an indicator matrix quantifying the opportunity any two members have to cluster together. (B) By dividing the connectivity and indicator matrices we produce the final consensus matrix which measures the frequency with which any two features cluster together.

The consensus clustering result is an (NxN) matrix of average feature connectivities generated from the clustering results of each iteration of the resampling scheme. It is calculated by dividing the number of times two features are found together in the same cluster by the number of times that they have been selected together in the sampling subsets. For each iteration, the clustering result is represented as a list where cluster membership is indexed against a feature identifier (e.g. gene id). We construct an (NxN) connectivity matrix from this list where we enter 1 if the features are in the the same cluster and 0 if they are not (Figure 1A). We also construct an (NxN) indicator matrix where we enter 1 if the features are present in the sampled subset and 0 if they are not. We then sum all of the connectivity and indicator matrices for iterations performed under the same conditions and divide the two to produce the consensus matrix (Figure 1B).

### Calculating cluster and membership robustness

We can use the consensus matrix ℳ to generate a measure of cluster robustness *m*(*k*) for each cluster *k*. First we create a set of indices *I_k _*for each cluster *k *indexed by the feature identifier and then calculate the average connectivity within each cluster. This is done by first summing the elements of the consensus matrix where members *i *and *j *are in the same cluster when *i *<*j *(as the matrix is symmetrical) and then dividing by the total number of entries in that half of the matrix (excluding the matrix diagonal) as given by equation 1.

(1) $$m(k)=\frac{1}{N_{k}(N_{k}-1)/2}\sum_{\begin{array}{l} i,j\in I_{k}\\ i<j \end{array}}ℳ(i,j)$$

We can also calculate the membership robustness *m_i_*(*k*) of each element of the data set *D *= {*e*_1..._*e_N_*} for each cluster *k *as the average connectivity between any one element *e_i _*and all of the other elements of the cluster. First we sum the average connectivities of all of the other members of the cluster to the current member *e_i _*(i.e. how often the other members are found with the current element in the current cluster) and divide by the number of other elements in the cluster as given by equation 2.

(2) $$m_{i}(k)=\frac{1}{N_{k}-1\{e_{i}\in I_{k}\}}\sum_{\begin{array}{l} j\in I_{k}\\ j\neq i \end{array}}ℳ(i,j)$$

It is important to note that the consensus matrix itself is not sufficient to calculate cluster and membership robustness. We need in addition to have *I_k _*for all clusters *K *where *k *∈ *K *which we generate by clustering the whole dataset under the same conditions as for the re-sampled data. This is the reference clustering result whose robustness we are testing in the re-sampling procedure. It is possible to use the consensus matrix itself as a distance matrix for clustering directly which would generate a cluster structure from which you could generate the index set *I_k _*for all clusters. In this paper we use the former approach as it allow us to explicitly define the distance method and clustering conditions used and to compare the re-sampled consensus directly to it.

### Merging consensus clustering results

Having developed methods for executing multi-algorithmic and multi-condition clustering for the *consensus clustering *approach of Monti et al. [14] we wanted to explore the effects of combining the consensus clustering results to see if these merged results mitigated for problems that can be associated with some types of clustering algorithms. For example, hierarchical clustering algorithms are highly sensitive to outliers whereas partitional ones tend to be relatively insensitive [23]. We implemented a straight forward approach *merged consensus clustering *whereby we used weighted averaging to combine the consensus matrices from clustering experiments using different algorithms and/or conditions while only merging results for experiments with the same number of clusters. This means that we produce a merged consensus matrix for each *k *value assessed during the resampling procedure. In the absence of additional prior information, equal weighting was applied to the combination of consensus matrices, but this can be specified directly by the user as a vector of weight values $\vec{w}$ = [0 - 1] if they wish to bias the merge towards any particular algorithm and/or parameter set. As with the original consensus clustering approach we need to provide a clustering structure in order to calculate cluster and membership robustness from a merged consensus matrix. We do this explicitly because each experimental condition can potentially produce a different clustering structure on which to assess robustness. When comparing a consensus result to a merge consensus result we use identical reference clustering structures to allow the effects of merging to be directly compared.

### Estimating the cluster number

The true cluster number (*k*) of a data set can be estimated by finding the value of *k *at which there is the greatest change in area under the cumulative density function (CDF) calculated from the consensus matrix across a range of possible values of *k*.

If we order the unique elements of a consensus matrix descending by value we can calculate a cumulative density function *CDF *(*c*) defined over the range *c *= 0[1] using equation 3.

(3) $$CDF(c)=\frac{\sum_{i<j}1\{ℳ(i,j)\leq c\}}{N(N-1)/2}$$

We can then calculate the area under the curve, *AUC *using equation 4.

(4) $$AUC=\sum_{i=2}^{m}\left[x_{i}-x_{i-1}\right]CDF(x_{i})$$

where *x_i _*is the current element of the *CDF *and *m *is the number of elements.

If every iteration of a consensus clustering experiment clusters the same features together (i.e. the clustering is perfectly consistent) then the consensus matrix elements will be either 1 or 0 and the resulting *AUC *= 1. By calculating the *AUC *for each consensus matrix we can quantitatively compare different clustering results and benchmark them against a perfectly consistent clustering result. We can extend this method to estimate the true cluster number *k *by *consensus clustering*, varying only the cluster number. We calculate the quantity Δ*K*, which is the change in *AUC *as we vary *k*, and define the optimal *k *value as that which coincides with a peak in Δ*K*.

### General procedure of a clusterCons analysis run

The *clusterCons *package takes as input the data to be clustered as a numeric matrix where each row is labelled with a unique identifier (e.g. gene id) and each column a unique condition identifier (e.g. patient id, time-point). The user then specifies the clustering algorithms to be used, either from a pre-defined set (*agnes*, *diana*, *k-means*, *pam*, *hclust*, *apcluster*) or user defined and, optionally, customised running parameters such as cluster number range, iteration number and sampling proportion. The package then carries out *consensus clustering *and returns consensus matrix objects for each specified set of algorithms and parameters. These consensus matrix objects can be used directly as distance matrices or to quantify cluster and membership robustness. The user can also specify whether they would like to generate a merge consensus matrix for each value of *k*. This merge matrix is generated by (weighted or un-weighted) averaging of the consensus matrices by providing an optional vector of weights $\vec{w}$ = [0 - 1] and is designed to mitigate for extremes in consensus values that can be created by the sensitivity of some algorithms to particular data structures. The merge consensus matrices can also be used as distance matrices themselves in new clustering experiments. They can also be used to re-calculate cluster and membership robustness using as reference the clustering structures produced by the original *consensus clustering *experiment.

When an estimation of the true cluster number is required, consensus clustering objects from a range of *k *values are used to calculate the *AUC *and Δ*K *values. The Δ*K *values are then plotted against *k *in a "*delta-K*" plot to identify the peaks visually and estimate the true value of *k*.

## Results and Discussion

### Implementation

*clusterCons *has been implemented with R version 2.10.0 as a package and successfully tested on Linux, Windows and Mac OS workstations. Execution times are dependent on the size, complexity and range of consensus runs and the power of the computer. As a guide, an example run with 45 clustering conditions, 100 iterations and a data matrix of 500 elements executed in 30 minutes on an entry level workstation (3 GB RAM, dual-core 1.60 GHz processors). Where faster execution times are needed it is possible to run *clusterCons *from within a batch script and each iteration farmed out as a separate process on a multi-processor facility. We routinely run larger experiments as batch arrays on the Edinburgh Array and Compute Data Facility (ECDF) a 1456 processor HPC compute cluster [24].

### Validation

#### Simulated expression data

**Figure 2:**
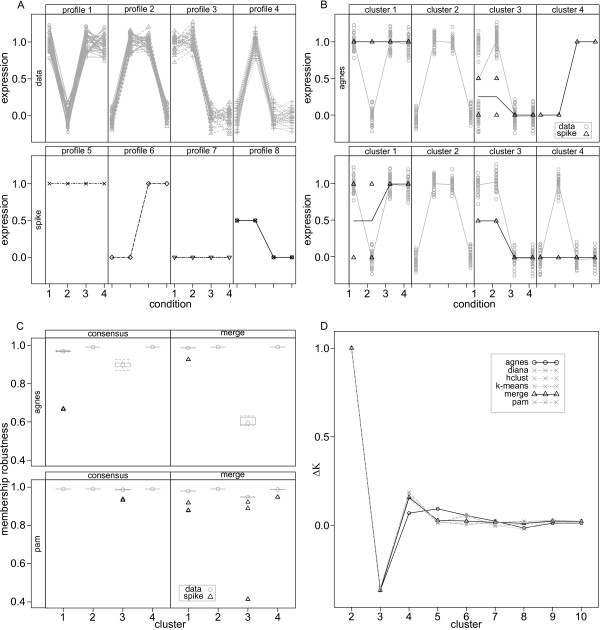
**Consensus clustering with simulated gene expression profile data**. (A) Simulated gene expression sets were generated randomly from normal distributions centred around four characteristic profiles (1,0,1,1), (0,1,1,0), (1,1,0,0), (0,1,0,0) which were then spiked with expression profiles centred on (1,1,1,1), (0,0,1,1), (1,1,0,0) and (0.5,0.5,0,0). (B) Unsupervised clustering with agnes and pam was used to partition the expression data into four clusters. Only profiles 1 and 2 were successfully identified by *agnes*, profiles 3 and 4 were consolidated and the (0,0,1,1) spike data segregated into a new profile (top row). Conversely, *pam *identified four expression profiles and segregated spike data into the closest matching profiles (bottom row). (C) Clustering with *agnes *and *pam *was repeated using *clusterCons *and the membership robustness calculated for each profile ('consensus' panels). For *agnes*, the spike data in cluster 1 are revealed as outliers (open triangles) and the robustness for cluster 3 is noticeably lower than clusters 1-4 reflecting its heterogeneity. Clusters produced by *pam *showed high robustness with only the spike data in cluster 3, observable as outliers (open triangles). Merge consensus matrices were generated from these two consensus clustering results and cast onto the *agnes *and *pam *clustering structures ('merge' panels) producing a more balanced view of membership (and hence cluster) robustness. For *agnes*, as expected, profiles 1, 2 and 4 remain largely unaffected, but profile 3 is heavily penalised as it is inconsistent between clustering algorithms. All *pam *profiles retain their high membership robustness, but now spike data are revealed for all profiles as outliers. (D) The optimal cluster number was estimated by finding the largest change in area under the cumulative density curve (AUC) for the consensus matrix of each clustering experiment by cluster number. Using this approach, the merge consensus matrix correctly predicted an optimal cluster number of 4, whereas *agnes *predicted 5.

We evaluated the performance of *clusterCons *with both simulated and experimental data sets. For simulated data sets, we generated expression data with four distinct expression profiles for 120 genes over four conditions (Figure 2A 'data' panels). Experimental gene expression data is noisy, so to assess how well standard and *consensus clustering *were able to classify the simulated profiles in the presence of noise we spiked the data with four noise profiles (Figure 2A 'spike ' panels). Clustering was then performed using the *agnes *(hierarchical) and *pam *(partitional) algorithms provided by the CRAN *package cluster *[25] using a euclidean distance metric and a fixed cluster number of four, the known number of true expression profiles. We wanted to determine if the algorithms could correctly identify the four original expression profiles and segregate the noisy data into the profiles that they most closely matched. Classification of the spiked profiles by *agnes *resulted in the fusion of profiles 3 and 4 into a new cluster and the creation of an additional cluster comprising one group of spiked profiles (Figure 2B 'agnes' panels). In contrast, the *pam *clustering algorithm correctly classified all profiles including the spiked ones (Figure 2B 'pam' panels). We then performed a *clusterCons *analysis using both algorithms (resampling proportion = 0.8, iterations = 100) and calculated the membership robustness for each cluster (Figure 2C 'consensus' panels). Membership robustness values from the *agnes *clustering show cluster 3, the fused cluster, to be the least robust, but notably the spiked data are not revealed as outliers since the cluster itself is heterogeneous. In addition the spiked data in cluster 1 are identified despite fitting the profile at 3 out of 4 positions (see Figure 2B 'agnes' cluster 1). This is a result of the high sensitivity of hierarchical clustering methods to outliers when the cluster is not generally heterogeneous [23]. As expected, clustering with *pam *produced very robust cluster membership, with only the more divergent of the spiked profiles of cluster 3 present as outliers (open triangles). This reflects the relative insensitivity of partitional clustering methods to outliers [23].

These opposing observations reveal a potential for significant errors in class discovery even with this simple data set. If we merge the consensus matrices resulting from the *pam *and *agnes clusterCons *runs into a single matrix by averaging them, we can then use the merge matrix to calculate cluster and membership robustness using either the *pam *or *agnes *clustering structures as a reference (Figure 2C 'merge' panels).

This effectively allows the user to blend the output of the two different clustering methodologies and provide a more balanced representation of the true robustness of the clustering schema and membership. We can see that without significantly affecting the membership robustness of the *pam *clustering (which correctly identified the 4 profiles) we now have a much improved estimate of membership robustness where all of the divergent spike profiles are weighted down (open triangles). Even more importantly, for the *agnes *clustering structure, cluster 3 (the fused cluster of profiles 3 and 4) is heavily penalised in terms of membership robustness. The ability to identify the minor outlier (only 1 of 4 measurements deviates from the true profile shape) in cluster 1 is retained, but diminished (as this is not identified by *pam*). This analysis shows that by applying a consensus merge methodology we can isolate outliers in a quantitative manner and assess how well different clustering algorithms partition the data.

In addition to calculating cluster and membership robustness, we used *clusterCons *to estimate the correct number of clusters in the data, by creating a "*delta-K*" plot [26] (Figure 2D). This plot was generated by running *clusterCons *with multiple algorithms over a range of possible cluster number values. Perfectly robust clustering generates consensus matrices with elements being either 1 or 0 as the same feature pairs are always found together in the same cluster. We created an empirical cumulative density plot from the value sorted elements of the consensus matrix and then calculated the area under the curve (*AUC*) which for perfect clustering is equal to 1. By calculating the change in the *AUC *as cluster number varies we identified which cluster number coincided with the greatest improvement in AUC and thus best estimated the cluster number. Figure 1D shows the "*delta-K*" plot of the run for each of five algorithms and for the merged consensus matrix. The inability of *agnes *to correctly classify the profiles is revealed as a prediction of *k *= 5 for the optimal cluster number. In contrast, all of the other matrices including the merge (which includes the *agnes *consensus matrix data) correctly predict *k *= 4.

We now apply our consensus clustering methodology to two biological problems: classifying leukaemia patients and identification of developmentally co-regulated genes using microarray data sets.

#### Classification of leukaemia patients

Classification and class discovery methods are commonly used to stratify patients into groups using either quantitative measures (e.g. gene expression, protein, metabolite levels) or indexed qualitative or semi-quantitative measures (e.g. symptoms, severity, treatment). Identifying disease associated genes provides an opportunity to improve diagnosis, treatment and understanding of the disease and has been widely used in oncology [27], neurology [28] and cardiology [29]. To test the utility of consensus merge clustering in patient class discovery using gene expression data we used the leukaemia gene expression data set of Golub et al. [22] which contains profiles of patients suffering from either acute myeloid leukaemia (*aml*) or acute lymphoblastic leukaemia (*all*). This data set is publicly available and is easily obtainable as an R data object within the CRAN hopach package [30].

**Figure 3:**
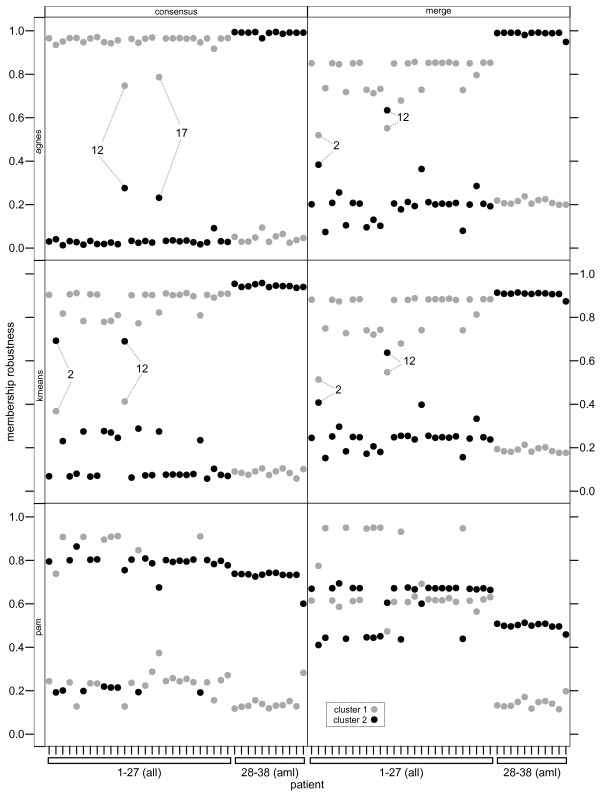
**Patient class discovery using a consensus clustering approach**. The leukaemia gene expression data set of Golub et al. [22] was used to assess the utility of consensus clustering to the segregation of patients into either an *all *(1-27) or *aml *(28-38) cluster. Consensus clustering was carried out with 500 iterations and the clustering algorithms *agnes*, *k-means *and *pam *and the membership robustness was calculated and plotted against patient number for both clusters. All three algorithms correctly segregated the *aml *patients into the same cluster ('consensus' panels, cluster 2, black filled circles), but only *agnes *(all) and *k-means *(all but 2 and 12) segregated the *all *patients reliably, whereas *pam *failed to correctly segregate 8/27 *all *patients. Merge consensus matrices were generated and membership robustness calculated for each of the three clustering structures ('merge' panels). *Agnes *and *k-means *produced almost identical results correctly segregating all patients apart from *aml *patients 2 and 12. *Pam *correctly segregated all *aml *patients, but could not segregate 19/27 *all *patients.

To reduce the scale of the clustering problem and exclude uninformative genes we selected the 200 genes that had the highest expression variance across patients. We performed a *clusterCons *run on the transposed expression matrix with 500 iterations and default parameters for the *agnes *and *pam *algorithms using a euclidean distance measure and with *k-means *using the MacQueen method [31] with *k *= 2 (as there are two clusters, *aml *and *all*) and calculated the membership robustness from both consensus and merge consensus matrices. Membership robustness was plotted against patient number for both clusters to visualise how well each algorithm segregated patients into one or other cluster with a perfect classification for any one patient being a membership robustness of 1 in one cluster and 0 in the other (Figure 3). The consensus clustering results obtained from each algorithm successfully assigned the *aml *patients (numbers 28-38) into the same cluster (Figure 3 'consensus' panels, cluster 2). Only *agnes *correctly segregated all of the *all *patient samples into the correct cluster, but highlighted patients 12 and 17 as having the lowest membership robustness in the cluster. Class discovery by *k-means *correctly segregated all but patients 2 and 12 into the *all *group. The *pam *algorithm incorrectly assigned patients 2,3,6,9-11,14 and 23 to the *aml *cluster. These results illustrate the benefit of *consensus clustering *compared to the discrete classes produced by standard clustering methods. Consensus clustering not only highlights the quality of the classification (i.e. the distance between membership robustness values for each patient for each cluster), but also allows a standardised and comparative framework to assess the relative performance of different clustering algorithms and conditions. In this case and for this data set the *pam *algorithm would be a poor choice as it is unable to segregate the data according to the known classes for 8 out of 27 patients.

We next assessed the performance of *merge consensus clustering *by averaging the consensus matrices produced by each of the algorithms into a single merge consensus matrix and then calculating the adjusted membership robustness values for each algorithm using the corresponding clustering structures from the original experiments (Figure 3 'merge' panels). This allows the direct comparison of membership robustness values for each patient between the consensus and merge consensus values as the same clustering structure is used to make the calculations for both. As further illustration of the unsuitability of *pam *to the classification of this data set we now see that merge consensus clustering produces almost identical membership robustness values for both clusters for 19 out of the 27 *all *patients. In contrast, the merge robustness plots for both *agnes *and *k-means *are almost identical and in both cases patients 2 and 12 fail to be robustly placed into either class (i.e. their membership robustness values for clusters 1 and 2 are very close to each other). This is crucial for the purposes of trying to determine whether the class of the patient has been reliably determined. Comparing the membership robustness values for patients 2 and 12 between merge and consensus plots we see that the averaging of the consensus matrices has pulled the values close to each other which increases our uncertainty about the correct class for these patients. In the absence of merge clustering (and in a real life situation where the class is not known before hand) we would have confidently assigned patients 2,12 and 17 to the *all *group in the case of *agnes *and patients 2 and 12 to the *aml *group in the case of *k-means*.

**Figure 4:**
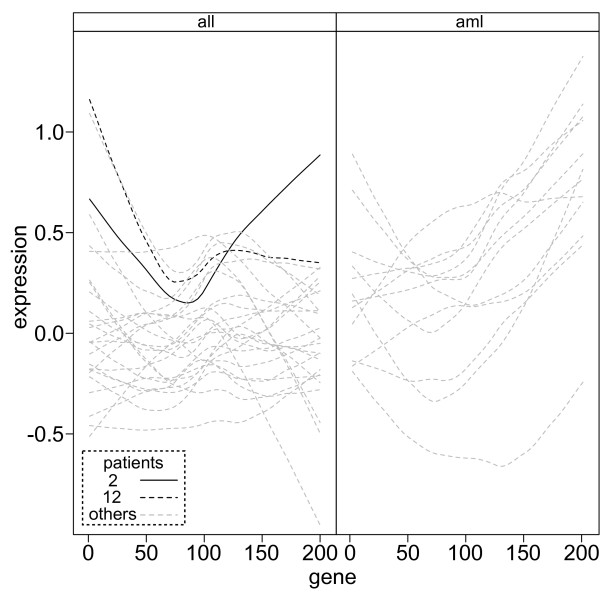
**Leukaemia patient expression profiles**. The expression profiles of all leukaemia patients were plotted against the gene identification number grouped by patient class ('all' and 'aml' panels) and the profiles of atypical *all *profiles for patients 2 (solid black line) and 12 (dashed black line) highlighted.

To assess whether the expression profiles of patients 2 and 12 are indeed unusual for *all *patients we plotted the patient expression profiles grouped by class (Figure 4). The expression profiles of both patients are atypical of the *all *group; patient 2 has very high expression levels for genes 1-50 and 130-200 and patient 12 has high expression for genes 1-40 and 160-200 in contrast to all other *all *patients. We have demonstrated the utility of both consensus and merge consensus clustering as a way of quantifying the robustness of patient classification, both in terms of selecting suitable algorithms and identifying patients that are atypical of their group.

#### Identifying expression modules during Drosophila peripheral nervous system development

Microarray gene expression experiments are increasingly being used to look at temporal expression patterns at the organism, tissue and cellular level during development [32-35] as well as other more general multi-condition scenarios. To illustrate the utility of applying a *merge consensus clustering *methodology to this type of problem we used *clusterCons *to classify gene expression profiles during the development of the peripheral nervous system (PNS) of the fruitfly, *Drosophila melanogaster*. We measured gene expression using the Affymetrix GeneChip Drosophila genome 2.0 array using mRNA isolated from flow sorted cell populations that were highly enriched in cells of the early PNS (TIS and APJ, data available from the gene expression omnibus (GEO) accession GSE21520). The data set covers four experimental conditions, three stages of PNS development in wild type flies (conditions 1-3) and one in flies mutant for the transcription factor atonal which is required for the normal formation of the PNS (condition 4) [36]. We wanted to identify groups of genes that shared discrete expression profiles across the four conditions as a starting point to discovering co-regulated genes. We first selected all probe-sets whose expression in cells of the early PNS were highly enriched compared to control cells in any of the conditions (fdr < = 1%, ratio > = 2).

This produced a list of 526 probe-sets. This list was further reduced to the 200 probe-sets that had the highest expression variance across conditions to maximise the chance of producing discrete and informative profiles. The final pre-classification transformation was to unitise the expression matrices so that the classification was made on the basis of the shape of the expression profile and not the magnitude.

**Figure 5:**
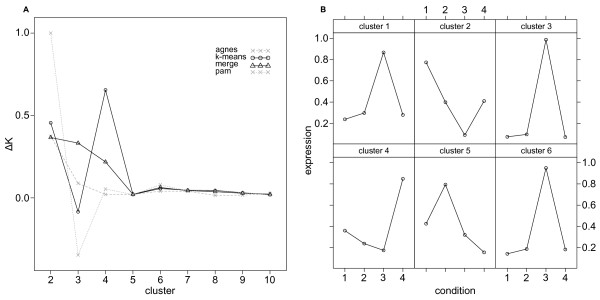
**Discovering gene expression profiles with consensus clustering**. A fruitfly PNS gene expression data set (TIS, APJ, data available from the gene expression omnibus (GEO) accession GSE21520) was used to test the ability of *clusterCons *to identify gene expression profiles across a developmental time-series. (A) Consensus clustering was performed with *agnes*, *pam *and *k-means *algorithms with 100 iterations and cluster numbers *k *= {2, 3...10}. The optimal cluster number was estimated by calculating first the AUC and then the delta-K values for the consensus and merge consensus matrices and a delta-K plot generated. The small, but consistent peak at *k *= 6 for *k-means*, *pam *and *merge *consensus matrices was select for further study using the *k-means *clustering structure. (B) Relative gene expression means were plotted for all probe-sets by cluster revealing discrete and stereotypical profiles describing stage and genotype specific features. Among these are profiles for early (clusters 2 and 4), mid (cluster 5) and late (clusters 1,3 and 6) expressed genes as well as differentiation of genes that are expressed lower (clusters 2 and 5) or higher (cluster 4) in the atonal mutant.

**Figure 6:**
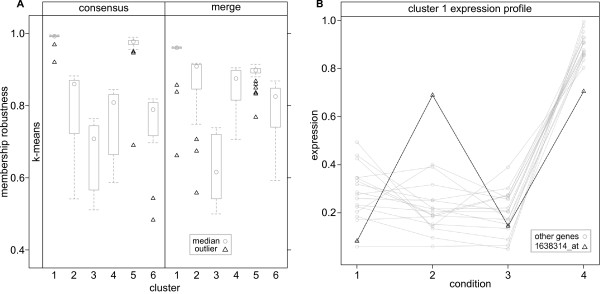
**Refining gene expression profiles with merge consensus clustering**. We compared the cluster and membership robustness of consensus and merge consensus clustering matrices using the *k-means *clustering structure. (A) For the consensus clustering results, clusters 1 and 5 were highly robust (*cr *= 0.99 and 0.97), clusters 2-4 and 6 were moderately robust (*cr *= 0.81, 0.66, 0.76 and 0.74) and outliers (open black triangles) were evident for clusters 1, 5 and 6. Refinement of the robustness measures by merge consensus clustering broadly maintained or improved the overall cluster robustness (*cr *= 0.93, 0.87, 0.63, 0.85, 0.90 and 0.79, clusters 1-6 respectively), but re-segregated the outliers for clusters 1,2,5 and 6. For example, a striking outlier appears for the highly conserved cluster 1 as a result of merge consensus clustering (probe-set 1638314_-_at, *mr *= 0.99 → 0.66). (B) This outlier is confirmed by plotting the relative gene expression for all of the probe-sets in cluster 1 (probe-set 1638314_-_at black line, open black triangles).

We performed a *clusterCons *run on the prepared data set with 100 iterations and the same parameters for the *agnes*, *k-means *and *pam *algorithms that were used previously. The cluster number, *k *was varied from 2-10 and the cluster and membership robustness, *AUC *and Δ*K *were calculated. The "*delta-K*" plot (Figure 5A) revealed a peak where *k *= 4 and a smaller peak where *k *= 6 with both *k-means *and *pam *and a peak only at *k *= 6 with the merge matrix. This is typical of gene expression clustering where there are often a low number of high order partitions in the data which can be further partitioned into smaller groups with fine underlying structure. In order to maximise the diversity of profiles and minimise the size of the clusters we chose to continue our analyses with *k *= 6 using the *k-means *reference clustering structure. We plotted the averaged expression values of the probe-sets classified into each cluster against condition (Figure 5B) revealing a set of highly distinct and stage specific profiles. Among these are profiles for genes expressed in early (clusters 2 and 4), mid (cluster 5) and late (clusters 1,3 and 6) PNS development. In addition there is differentiation between genes that are expressed in the atonal mutant at a lower (clusters 2 and 5) or higher (cluster 4) level. We next compared the membership robustness between consensus and merge consensus clustering matrices. The consensus results show that clusters 1 and 5 are highly robust (cluster robustness *cr *= 0.99 and 0.97 respectively) in comparison to clusters 2-4 and 6 (*cr *= 0.81, 0.66, 0.76 and 0.74, respectively). The most extreme outlier for cluster 1 has a membership robustness *mr *= 0.92 (Figure 6A 'consensus' panel) which is highly robust under this clustering regime. Cluster 5 has a more pronounced outlier with *mr *= 0.69, whereas clusters 2-4 have no outliers, but broad inter-quartile ranges (*IQR *= 0.14, 0.14 and 0.13, respectively). Cluster 6 has two definite outliers with *mr *= 0.54 and 0.48.

These results allow us to rank clusters based on their stability during re-sampling, prioritise expression profiles and rank cluster members by their robustness values. We can prioritise genes to include for further investigation and those to eliminate based on low stability within the cluster providing a quantitative method to remove cluster members reducing noise within the profile. We completed our analysis by casting the merge matrix produced from consensus clustering with all three algorithms onto the *k-means *clustering structure and calculating cluster and membership robustness. The merge clustering produced an improvement in the membership robustness of clusters 2,4 and 6 (*mr *= 0.87, 0.85 and 0.79) and only a small reduction in those of clusters 1 and 5 (*mr *= 0.93 and 0.90), whereas cluster 3 was essentially unaffected (*mr *= 0.63). The refinement of the consensus clustering results by merging results in significant changes in the membership robustness of several probe-sets (Figure 6B). Notably, in cluster 1 there is now an outlier with *mr *= 0.66 (probe-set 1638314 at, previous *mr *= 0.99). To determine the basis for this change we plotted the expression profiles for all probe-sets in cluster 1 (Figure 6B). In this plot, probe-set 1638314_-_at (black line, open triangle) has a very different relative expression level at condition 2. Similar analysis across the other merge clustering results revealed that merge clustering not only identified outliers when single algorithm consensus clustering did not, but also re-classified some probe-sets as not being outliers. For example, in cluster 5 (Figure 5A) the most extreme outlier had its membership robustness increase from *mr *= 0.69, with consensus clustering, to *mr *= 0.77, with merge consensus clustering, despite a corresponding drop in cluster robustness from *cr *= 0.97 to *cr *= 0.90. Also for cluster 6 the two most extreme outliers showed decreases in membership robustness from *mr *= 0.54 to *mr *= 0.48 and *mr *= 0.70 to *mr *= 0.59 respectively with little change in cluster robustness (*cr *= 0.74 to *cr *= 0.79).

In this example we used *merge consensus clustering *to refine *consensus clustering *results to better represent the stability of the co-expressed gene clusters (improved estimation of cluster robustness) and identify ill fitting or 'noisy' members of the profiles (improved estimation of membership robustness).

Together these refinements provide the opportunity to quantify the performance of clusters and members using a hybrid approach that takes advantage of the classification features of different clustering algorithms. This allows for the prioritisation of clusters (profiles) and elements (probe-sets) in a quantitative rather than qualitative manner and is a framework for filtering clustering results to maximise the signal to noise ratio.

## Conclusions

We have extended the *consensus clustering *approach of Monti et al. [14] to allow *merge consensus clustering *and demonstrated its use with simulated and real gene expression data sets. We find that *merge consensus clustering *is effective in integrating consensus clustering results in a way that helps in the refinement of data classification and the identification of outliers. Crucially, this approach aids the selection of appropriate clustering algorithms and parameters and mitigates for the differential sensitivities of clustering algorithms to different data structures. Although we have demonstrated the benefit of *merge consensus clustering *for classifying gene expression data, it can be used to classify any data that can be represented numerically and should prove useful in the refinement and quantitative assessment of classification problems in general.

We have implemented *merge consensus clustering *as an R package, *clusterCons*. The package is fully documented, simple to use, freely available from the Comprehensive R package Archive Network (CRAN) [37] and easy to install using the resident package handling tools of R. We also include the latest version of the software and a user guide as additional files with this article (see Additional files 1 and 2, respectively). It provides methods to perform *consensus clustering *using any number of clustering algorithms and parameters. The resulting consensus matrices can be used as corrected distance matrices, to calculate cluster and membership robustness, estimate the optimal cluster number and to generate visualisations of the clustering structures. The *merge consensus clustering *approach extends the use of consensus matrices to integrate the results of consensus clustering experiments.

## Availability and Requirements

Project name: clusterCons

Project home page: http://sourceforge.net/projects/clustercons

Operating system(s): Platform independent

Programming language: R

License: GNU GPL

Any restrictions to use by non-academics: none

## Authors' contributions

TIS wrote the *clusterCons *package and performed all of the analyses. JDA and APJ supervised the work, and all authors contributed to the preparation of the final manuscript.

## Supplementary Material

Additional file 1 **The current *clusterCons *R package release**. The file includes the latest clusterCons package release with example scripts and full documentation. Click here for file

Additional file 2 **The clusterCons User Guide**. The file is a PDF document describing the use of clusterCons with examples for each function. Click here for file
