# Supplementary material for: Merged consensus clustering to assess and improve class discovery with microarray data
Source: BMC Bioinformatics. 2010 Dec 3;11:590. doi: 10.1186/1471-2105-11-590 (PMC3002369; doi:10.1186/1471-2105-11-590)
Supplement: Additional file 2 — The clusterCons User Guide. The file is a PDF document describing the use of clusterCons with examples for each function. [file 1471-2105-11-590-S2.PDF]

# Package ‘clusterCons’

October 12, 2010

**Type** Package

**Version** 1.0

**Title** Calculate the consensus clustering result from re-sampled clustering experiments with the option of using multiple algorithms and parameter

**Date** 2010-10-12

**Author** Dr. T. Ian Simpson, University of Edinburgh

**Maintainer** Dr. T. Ian Simpson <ian.simpson@ed.ac.uk>

**Depends** methods,cluster,lattice,RColorBrewer,grid,apcluster

**Suggests** latticeExtra

**Enhances** cluster

**Description** clusterCons is a package containing functions that generate robustness measures for clusters and cluster membership based on generating consensus matrices from bootstrapped clustering experiments in which a random proportion of rows of the data set are used in each individual clustering. This allows the user to prioritise clusters and the members of clusters based on their consistency in this regime. The functions allow the user to select several algorithms to use in the re-sampling scheme and with any of the parameters that the algorithm would normally take.

**License** GPL

**LazyLoad** yes

**URL** http://sourceforge.net/projects/clustercons/

## R topics documented:

clusterCons-package . . . . . 2
auc . . . . . 4
auc-class . . . . . 6
aucplot . . . . . 6
checks . . . . . 7
clrob . . . . . 8
cluscomp . . . . . 9
consmatrix-class . . . . . 11

data . . . . . . . . . . . . . . . . . . . . . . . . . . . . . . . . . . . . . . . . . . . 13
deltak . . . . . . . . . . . . . . . . . . . . . . . . . . . . . . . . . . . . . . . . . . 14
dk-class . . . . . . . . . . . . . . . . . . . . . . . . . . . . . . . . . . . . . . . . . 15
dkplot . . . . . . . . . . . . . . . . . . . . . . . . . . . . . . . . . . . . . . . . . . 16
expressionPlot . . . . . . . . . . . . . . . . . . . . . . . . . . . . . . . . . . . . . 17
expSetProcess . . . . . . . . . . . . . . . . . . . . . . . . . . . . . . . . . . . . . 18
membBoxPlot . . . . . . . . . . . . . . . . . . . . . . . . . . . . . . . . . . . . . . 18
memrob . . . . . . . . . . . . . . . . . . . . . . . . . . . . . . . . . . . . . . . . . 19
memroblist-class . . . . . . . . . . . . . . . . . . . . . . . . . . . . . . . . . . . 20
memrobmatrix-class . . . . . . . . . . . . . . . . . . . . . . . . . . . . . . . . . 21
mergematrix-class . . . . . . . . . . . . . . . . . . . . . . . . . . . . . . . . . . 22
wrappers . . . . . . . . . . . . . . . . . . . . . . . . . . . . . . . . . . . . . . . . 23

**Index** **25**

---

`clusterCons-package`

*Calculate consensus clustering results from re-sampled clustering experiments with the option of using multiple algorithms and parameters*

---

## Description

clusterCons is a package containing functions that generate robustness measures for clusters and cluster membership based on generating consensus matrices from bootstrapped clustering experiments in which a random proportion of rows of the data set are used in each individual clustering. This allows the user to prioritise clusters and the members of clusters based on their consistency in this regime. The functions allow the user to select several algorithms to use in the re-sampling scheme and with any of the parameters that the algorithm would normally take.

## Details

| | |
|---|---|
| Package: | clusterCons |
| Type: | Package |
| Version: | 1.0 |
| Date: | 2010-10-12 |
| License: | GPL |
| LazyLoad: | yes |
| Depends: | methods,cluster,lattice,RColorBrewer,grid,apcluster |
| Extends: | cluster |
| Suggests: | latticeExtra |

The user should first prepare an entirely numeric `data.frame` in which the conditions to be clustered are the column names and the unique ids of the entities are the row names. Compatibility of the resulting data.fram can be checked by using the `data_check` function.

## Functions to run the consensus clustering and retrieve robustness information

`cluscomp` - generate consensus matrices from re-sampled clustering experiments with the option of multiple algorithms and parameters
`clrob` - calculate the robustness of the clusters from the consensus matrix

memrob - calculate the cluster membership robustness from the consensus matrix

## Internal functions to call the individual clustering algorithms

agnes_clmem - wrapper for the agnes function of package cluster
diana_clmem - wrapper for the diana function of package cluster
hclust_clmem - wrapper for the hclust function of package cluster
kmeans_clmem - wrapper for the kmeans function of package cluster
pam_clmem - wrapper for the pam function of package cluster
apcluster_clmem - wrapper for the apclusterK function of package apcluster

## Functions to calculate AUC related metrics

auc - calculates the area under the curve for a series of clustering experiments with the same cluster number
aucs - calculates the areas under the curves of a series of clustering experiments over a range of cluster numbers
deltak - calculates the change in the area under the curve

## Functions to check data and object validity

data_check - check that the provided data.frame is formatted correctly
expSetProcess - extracts the data set from an object of class expressionSet
validConsMatrixObject - check the validity of a consmatrix object
validMergeMatrixObject - check the validity of a mergematrix object
validMemRobListObject - check the validity of a membership robustness list object
validMemRobMatrixObject - check the validity of a membership robustness matrix object
validAUCObject - check the validity of an "auc" class object
validDkObject - check the validity of an "dk" class object

## Functions to plot out performance curves

aucplot - plot area under the curve (AUC) plots from consensus clustering results
dkplot - plot change in AUC by cluster number (delta-K plot)
expressionPlot - plot the original data partitioned by cluster membership
membBoxPlot - plot a box and whisker plot of the membership robustness for each cluster

## Keywords

cluster

## See Also

cluster,lattice,apcluster

### Examples

#load data data(sim_profile);

#perform consensus clustering cmr <- cluscomp(sim_profile,algo=list('agnes','pam','kmeans'),clmin=2,clmax=7,rep=1[illegible]

#see the consensus and merge matrices summary(cmr);

#fetch the cluster robustness for agnes consensus clustering with k=3 clrob(cmr$e1_agnes_k3);

#show the membership robustness for cluster 1 memrob(cmr$e1_agnes_k3)$cluster1

#show the same, but for the merge against the k=3 agnes clustering structure #note we provide the reference matrix (which is the original cluster membership for agnes where k=3) clrob(cmr$merge_k3,cmr$e1_agnes_k3[illegible] memrob(cmr$merge_k3,cmr$e1_agnes_k3@rm)$cluster1;

#calculate the AUCs acs <- aucs(cmr);

#plot the AUC curves aucplot(acs);

#calculate the delta-Ks dks <- deltak(acs);

#plot the delta-K curves dkplot(dks);

#plot the expression profiles expressionPlot(sim_profile,cmr$e1_agnes_k3);

#plot the bwplot of membership robustness for the same membBoxPlot(memrob(cmr$e1_agnes_k3));

### Author(s)

Dr. T. Ian Simpson `<ian.simpson@ed.ac.uk>`

### References

Merged consensus clustering to assess and improve class discovery with microarray data. Simpson, T.I., Armstrong, J.D. and Jarman A.P. (submitted)

Consensus clustering: A resampling-based method for class discovery and visualization of gene expression microarray data. Monti, S., Tamayo, P., Mesirov, J. and Golub, T. Machine Learning, 52, July 2003.

---

## `auc` *Calculate area under the curve statistics*

---

### Description

These functions calculate the area under the curve (AUC) for cumulative density functions of a consensus matrix. The function `auc` operates on an indvidual consensus matrix whereas `aucs` operates on an entire `cluscomp` analysis result as described below.

### Usage

```
auc(x)
aucs(x)
```

## Arguments

| | |
|---|---|
| `x` | For `auc(x)`, provide a numeric square data matrix such as an individual consensus matrix. For `aucs(x)` provide a list of `"consmatrix"` class objects (see `consmatrix-class for details`) such as those produced directly by the `cluscomp` function.<br>The functions will not allow any missing values (NAs). |

## Value

`auc(x)` returns an individual AUC value.

`aucs(x)` returns a data.frame with the following variables.

| | |
|---|---|
| `k` | cluster number as a factor |
| `a` | algorithm identifier as a factor |
| `aucs` | the AUC value |

## Author(s)

Dr. T. Ian Simpson `<ian.simpson@ed.ac.uk>`

## References

Merged consensus clustering to assess and improve class discovery with microarray data. Simpson, T.I., Armstrong, J.D. and Jarman A.P. (submitted).

## See Also

`consmatrix-class`

## Examples

```
#load up a test cluscomp result
data('testcmr');

#look at the result structure
summary(testcmr);

#reduce size for the example
testcmr <- testcmr[1:12];

#calculate an individual AUC value for a consensus matrix
ac <- auc(testcmr$e1_kmeans_k2@cm);

#calculate all of the AUC values from the \code{cluscomp} result
acs <- aucs(testcmr);
```

## auc-class *Class "auc"*

### Description

Objects of class `'auc'` contain a data.frame which have three variables `k`, `a` and `auc` as described in the `aucs` function description. This class simply holds the result from a call to `aucs`.

### Objects from the Class

Objects can be created by calls of the form `new("auc", ...)`, although they are normally generated internally by the `aucs` function.

### Author(s)

Dr. T. Ian Simpson `<ian.simpson@ed.ac.uk>`

### References

Merged consensus clustering to assess and improve class discovery with microarray data. Simpson, T.I., Armstrong, J.D. and Jarman A.P. (submitted).

### See Also

Also see the `aucs` function.

### Examples

```
showClass("auc")
```

## aucplot *Generate an area under the curve plot using lattice graphics*

### Description

This function uses the `lattice` function `xyplot` to generate an AUC plot from a valid `"auc"` class object (see `auc-class`).

### Usage

```
aucplot(x)
```

### Arguments

| | |
|---|---|
| `x` | a valid "auc" class object (see `auc-class`), normally generated by the `aucs` function. |

### Author(s)

Dr. T. Ian Simpson `<ian.simpson@ed.ac.uk>`

### References

Merged consensus clustering to assess and improve class discovery with microarray data. Simpson, T.I., Armstrong, J.D. and Jarman A.P. (submitted).

### See Also

`consmatrix-class`

### Examples

```
#load up a test cluscomp result
data('testcmr');

#look at the result structure
summary(testcmr);

#reduce size for the example
testcmr <- testcmr[1:12];

#calculate all of the AUC values from the cluscomp result
acs <- aucs(testcmr);

#plot the AUC curve
aucplot(acs);
```

---

`checks` *Functions to check the integrity of various objects*

---

### Description

These methods are mainly internal although the user may like to check their original data using `data_check` before they perform consensus clustering experiments.

### Usage

```
data_check(x)
validConsMatrixObject(object)
validMemRobListObject(object)
validMemRobMatrixObject(object)
validMergeMatrixObject(object)
validAUCObject(object)
validDkObject(object)
```

### Arguments

| | |
|---|---|
| `x` | The data.frame object to be checked prior to using with the `cluscomp` function. |
| `object` | The object to be checked with the suitable function by type. These are used internally by several of the functions in the package. |

### Value

returns `TRUE` if check is passed or an error message if it is not

### Author(s)

Dr. T. Ian Simpson <ian.simpson@ed.ac.uk>

### References

Merged consensus clustering to assess and improve class discovery with microarray data. Simpson, T.I., Armstrong, J.D. and Jarman A.P. (submitted).

### Examples

```
#load data
data(sim_profile);

#check if this can be used by cluscomp
data_check(sim_profile);

#perform a clusomp run
cmr <- cluscomp(sim_profile,clmin=2,clmax=2,rep=10);

#check one of the consensus matrices
validConsMatrixObject(cmr$e1_kmeans_k2)
```

---

## clrob — *Calculate the cluster robustness from consensus clustering results*

---

### Description

This function calculates the cluster robustness from a `consmatrix` or `mergematrix` class object.

### Usage

```
clrob(x,rm)
```

### Arguments

| | |
|---|---|
| `x` | either a `consmatrix` or `mergematrix` object. |
| `rm` | (optional) if a `mergematrix` object is passed then you must provide a reference clustering structure to calculate cluster robustness against. These structures are stored with every `consmatrix` object in the 'rm' slot. You would normally select a reference matrix for a cluster number matching that of the `mergematrix` (see example below). |

### Value

Returns a data.frame of the cluster robustness values indexed by cluster number.

### Author(s)

Dr. T. Ian Simpson <ian.simpson@ed.ac.uk>

## References

Merged consensus clustering to assess and improve class discovery with microarray data. Simpson, T.I., Armstrong, J.D. and Jarman A.P. (submitted).

## See Also

Also see cluscomp, consmatrix and mergematrix.

## Examples

```
#load cmr (consensus clustering result produced by cluscomp)
data(testcmr);

#calculate the cluster robustness of the consensus matrix for pam where k=4
clrob(testcmr$e1_kmeans_k4);

#calculate the cluster robustness of the merge matrix in reference to the clustering stru
clrob(testcmr$merge_k4,testcmr$e1_kmeans_k4@rm);
```

---

# cluscomp — *Perform consensus clustering with the option of using multiple algorithms and parameters and merging*

---

## Description

Calculates an NxN consensus matrix for each clustering experiment performed where each entry has a value between 0 (never observed) and 1 (always observed)
When running with more than one algorithm or with the same algorithm and multiple conditions a consensus matrix will be generated for each. These can optionally be merged into a mergematrix by cluster number by setting merge=1.

## Usage

```
cluscomp(x, diss=FALSE, algorithms = list("kmeans"), alparams = list(), alweight
         clmin = 2, clmax = 10, prop = 0.8, reps = 50, merge = 0)
```

## Arguments

| | |
|---|---|
| x | data.frame of numerical data with conditions as the column names and unique ids as the row names. All variables must be numeric. Missing values(NAs) are not allowed. Optionally you can pass a distance matrix directly, in which case you must ensure that the distance matrix is a data.frame and that the row and column names match each other (as the distance matrix is a pair-wise distance calculation). |
| diss | set to TRUE if you are providing a distance matrix, default is FALSE |
| algorithms | list of algorithm names which can be drawn from 'agnes','diana','pam','kmeans' or 'hclust'. The user can also write a simple wrapper for any other clustering method (see details) |
| alparams | list of algorithm paramter lists using the same specification as for the individual algorithm called (see details) |

| | |
|---|---|
| `alweights` | list of integer weights for each algorithm (only used when merging consensus results between algorithms) |
| `clmin` | integer for the smallest cluster number to consider |
| `clmax` | integer for the largest cluster number to consider |
| `prop` | numeric for the proportion of rows to sample during the process. Must be between 0 and 1 |
| `reps` | integer for the number of iterations to perform per clustering |
| `merge` | an integer indicating whether you also want the merged matrices (1) or just the consensus ones (0), accepts only 1 or 0. |

## Details

`cluscomp` is an implementation of a consensus clustering methodology first proposed by Monti et al. (2003) in which the connectivity between any two members of a data matrix is tested by resampling statistics. The principle is that by only sampling a random proportion of rows in the data matrix and performing many clustering experiments we can capture information about the robustness of the clusters identified by the full unsampled clustering result.

For each re-sampling experiment run a zero square matrix is created with identical rows and columns matching the unique ids of the rows of the data matrix, this matrix is called the connectivity matrix. A second identically sized matrix is created to count the number of times that any pair of row ids are called in any one re-sampled clustering. This matrix is called the identity matrix. For each iteration within the experiment the rows sampled are recorded in the identity matrix and then the co-occurrence of all pairs are recorded in the connectivity matrix. These values are incremented for each iteration until finally a conensensus matrix is generated by dividing the connectivity matrix by the identity matrix.

The consensus matrix is the raw output from `cluscomp` implemented as a class `consmatrix`. If the user has specified to return a merged matrix in addition to the consensus matrices then for each clustering with the same k (cluster number value) an object of class `mergematrix` is also returned in the list which is identical to a `consmatrix` with the exception that the 'cm' slot is occupied by the merged matrix (a weighted average of all the consensus matrices for the cluster number matched consensus matrices) and there is no reference matrix slot (as there is no reference clustering for the merge). The user should instead call the `memrob` function using the merge matrix and providing a reference matrix from one of the cluster number matched `consmatrix` objects from which the merge was generated. This provides a way to quantify the difference between single and multi-algorithm resampling schemes.

## Value

a list of objects of class `consmatrix` and (if merge specified) `mergematrix`. See `consmatrix` and `mergematrix` for details.

## Author(s)

Dr. T. Ian Simpson `<ian.simpson@ed.ac.uk>`

## References

Merged consensus clustering to assess and improve class discovery with microarray data. Simpson, T.I., Armstrong, J.D. and Jarman A.P. (submitted)

Consensus clustering: A resampling-based method for class discovery and visualization of gene expression microarray data. Monti, S., Tamayo, P., Mesirov, J. and Golub, T. Machine Learning, 52, July 2003.

## See Also

cluster,clrob,memrob

## Examples

```
#load test data
data(sim_profile);

#perform a group of re-sampling clustering experiments accepting default parameters
#for the clustering algorithms
cmr <- cluscomp(sim_profile,algorithms=list('kmeans','pam'),merge=1,clmin=2,clmax=5,reps=

#simple example
cmr <- cluscomp(sim_profile,clmin=2,clmax=5,prop=0.8,reps=5)

#more complex example
alp <- list(method='complete')
cmr <- cluscomp(sim_profile,algorithms=list('agnes','pam'),alparams=list(alp,list()),clmi

#even more complex example
pamp <- list(metric='manhattan')
cmr <- cluscomp(sim_profile,algorithms=list('agnes','pam'),alparams=list(alp,pamp),alweig

#display resulting matrices contained in the consensus result list
summary(cmr);

#display the cluster robusteness for the kmeans k=4 consensus matrix
clrob(cmr$e2_pam_k4);

#plot a heatmap of the consensus matrix, note you access the cluster matrix object
#through the cm slot
heatmap(cmr$e2_pam_k4@cm);

#display the membership robustness for kmeans k=4 cluster 1
memrob(cmr$e2_pam_k4)$cluster1;

#merged consensus example
data(testcmr);

#calculate the membership robustness for the merge matrix when cluster number k=4, in ref
mr <- memrob(testcmr$merge_k4,testcmr$e1_kmeans_k4@rm);

#show the membership robustness for cluster 1
mr$cluster1;
```

---

consmatrix-class *Class "consmatrix"*

---

### Description

Objects of class 'consmatrix' are created to hold the results of a consensus clustering experiment along with the necessary ancillary data to allow the subsequent downstream calculations such as cluster and membership robustness. In addition the object holds the original call made when running cluscomp.

### Objects from the Class

Objects can be created by calls of the form new("consmatrix", ...), but are normally created internally by the cluscomp function to store consensus matrices and their associated metadata.

### Slots

- cm: Object of class "matrix" - the consensus matrix itself
- rm: Object of class "data.frame" - the cluster membership of the full (i.e. not consensus) clustering result when the current algorith is called with the same algorithm parameters as the consensus clustering run. This is needed to be able to work with merge matrices that need a clustering structure on which to operate to produce cluster and membership robustness values.
- a: Object of class "character" - the clustering algorithm name
- k: Object of class "numeric" - the cluster number (k) used
- call: Object of class "call" - the original parameters passed to cluscomp for provenance and reproducibility

### Author(s)

Dr. T. Ian Simpson <ian.simpson@ed.ac.uk>

### References

Merged consensus clustering to assess and improve class discovery with microarray data. Simpson, T.I., Armstrong, J.D. and Jarman A.P. (submitted).

### See Also

See Also cluscomp

### Examples

```
showClass("consmatrix");

#you can access the slots in useful ways

#load a cmr
data(testcmr);

#get a consensus clustering matrix via the 'cm' slot
cm <- testcmr$e1_kmeans_k4@cm;

#this can be used as a distance matrix, e.g. for a heatmap
heatmap(cm);

#or as a new distance matrix
```

```
dm <- data.frame(cm) #first convert to a data.frame
#make sure names are the same for rows and columns
names(dm) <- row.names(dm);

#you need to explicitly tell cluscomp that you are passing a distance matrix
cmr2 <- cluscomp(dm,diss=TRUE,clmin=2,clmax=4,rep=2);

#for merge consensus clustering you take advantage of the reference matrix (rm) slot
#cluster robustness for agnes with cluster number (k) = 3
clrob(testcmr$merge_k3,testcmr$e1_kmeans_k3@rm);
#membership robustness for cluster 1
memrob(testcmr$merge_k3,testcmr$e1_kmeans_k3@rm)$cluster1;
```

---

data *Data sets for the clusterCons package*

---

## Description

These data sets are used by the examples in the package function descriptions and allow the user to explore the functionality of the package

## Usage

```
data(golub);
data(sim_class);
data(sim_profile);
data(testcmr);
```

## Format

golub : data.frame of gene expression values for 999 genes for 38 leukemia patients (1-27) ALL and (28-38) AML. sim_class : data.frame of 200 simulated gene expression values for 30 conditions where there are 4 discrete classes of expression profile, for testing clustering with the transposed data (clustering by column). sim_profile : data.frame of 120 simulated gene expression values for 4 conditions where there are 4 discrete classes of expression profile, for testing general clustering (clustering by row). testcmr : list of consensus and merge matrix results from a `cluscomp` run (see `consmatrix-class` and `mergematrix-class`).

## Author(s)

Dr. T. Ian Simpson <ian.simpson@ed.ac.uk>

## References

Merged consensus clustering to assess and improve class discovery with microarray data. Simpson, T.I., Armstrong, J.D. and Jarman A.P. (submitted).

Molecular classification of cancer: class discovery and class prediction by gene expression monitoring. Golub, TR and Slonim, DK and Tamayo, P and Huard, C and Gaasenbeek, M and Mesirov, JP and Coller, H and Loh, ML and Downing, JR and Caligiuri, MA and Bloomfield, CD and Lander, ES. Science 1999, 286:531-537

### Examples

```
#cluster by class
data(sim_class);
cutree(agnes(t(sim_class)),4);

#cluster by profile
data(sim_profile);
cutree(agnes(sim_profile),4);
```

---

## deltak — *Function to calculate the change in the area under the curve (AUC) across a range of cluster number values*

---

### Description

This function takes an "auc" class object and calculates the difference in AUC value by cluster number (called delta-K). Peaks in delta-K coincide with the cluster numbers that are most robust and provide estimates for the optimal cluster number.

### Usage

```
deltak(x)
```

### Arguments

| | |
|---|---|
| x | a valid "auc" class object, normally provided as a result from the aucs function. |

### Value

deltak(x) returns a data.frame with the following variables.

| | |
|---|---|
| k | cluster number as a factor |
| a | algorithm identifier as a factor |
| dk | the delta-K value |

### Author(s)

Dr. T. Ian Simpson <ian.simpson@ed.ac.uk>

### References

Merged consensus clustering to assess and improve class discovery with microarray data. Simpson, T.I., Armstrong, J.D. and Jarman A.P. (submitted).

### See Also

Also see the aucs function.

## Examples

```
#load a test cluscomp result set
data(testcmr)

#reduce size for the example
testcmr <- testcmr[1:12];

#calculate the AUCs
acs <- aucs(testcmr);

#calculate the delta-K values
dks <- deltak(acs);
```

---

dk-class *Class "dk"*

---

## Description

Objects of class 'dk' contain a data.frame which have three variables k, a and deltak as described in the deltak function description. This class simply holds the result from a call to deltak.

## Objects from the Class

Objects can be created by calls of the form `new("dk", ...)`, although they are normally generated internally by the deltak function.

## Author(s)

Dr. T. Ian Simpson <ian.simpson@ed.ac.uk>

## References

Merged consensus clustering to assess and improve class discovery with microarray data. Simpson, T.I., Armstrong, J.D. and Jarman A.P. (submitted).

## See Also

Also see the aucs function.

## Examples

```
showClass("dk")
```

dkplot — *Generate a delta-K plot from area under the curve (AUC) values across multiple cluster numbers.*

## Description

This function uses the `lattice` function `xyplot` to generate an delta-K plot from a valid "`dk`" class object (see `dk-class`).

## Usage

```
dkplot(x)
```

## Arguments

| | |
|---|---|
| x | a valid "dk" class object (see `dk-class`), normally generated by the `deltak` function. |

## Author(s)

Dr. T. Ian Simpson <ian.simpson@ed.ac.uk>

## References

Merged consensus clustering to assess and improve class discovery with microarray data. Simpson, T.I., Armstrong, J.D. and Jarman A.P. (submitted).

## See Also

`consmatrix-class`

## Examples

```
#load up a test cluscomp result
data('testcmr');

#look at the result structure
summary(testcmr);

#reduce size for the example
testcmr <- testcmr[1:12];

#calculate all of the AUC values from the cluscomp result
acs <- aucs(testcmr);

#calculate all of the delta-K values
dks <- deltak(acs);

#plot the delta-K curve
dkplot(dks);
```

`expressionPlot` *Generate a profile plot for the data partitioned by cluster membership.*

## Description

This function uses the `lattice` function `xyplot` to generate a profile plot of the data values grouped by cluster in a multi-panel plot. The function takes as input the original data.frame() and a valid `"consmatrix"` class object (see `consmatrix-class`) by which to segregate the data.

## Usage

```
expressionPlot(x,cm);
```

## Arguments

| | |
|---|---|
| `x` | the original data.frame() object used in the clustering. |
| `cm` | a valid `"consmatrix"` class object generated by the `cluscomp` function. |

## Author(s)

Dr. T. Ian Simpson `<ian.simpson@ed.ac.uk>`

## References

Merged consensus clustering to assess and improve class dicovery with microarray data. Simpson, T.I., Armstrong, J.D. and Jarman A.P. (submitted).

## See Also

`consmatrix-class`

## Examples

```
#load up the data set
data(sim_profile);

#load up an example cluscomp result with this data
data('testcmr');

#plot the expression profiles
expressionPlot(sim_profile,testcmr$e1_kmeans_k4);
```

---

`expSetProcess` *Internal function to extract the data from an expressionSet class object from the affy package for use with cluscomp*

---

### Description

This is a convenience function that is used internally to allow the user to pass an expressionSet object from the microarray processing package 'affy' directly to the `cluscomp` function.

### Usage

```
expSetProcess(x)
```

### Arguments

| | |
|---|---|
| `x` | An object of class expressionSet from the Bioconductor package 'affy'. |

### Value

when called directly, returns a suitably labeled data.frame() object of the expressionSet expression values.

### Author(s)

Dr. T. Ian Simpson `<ian.simpson@ed.ac.uk>`

### References

Merged consensus clustering to assess and improve class discovery with microarray data. Simpson, T.I., Armstrong, J.D. and Jarman A.P. (submitted).

---

`membBoxPlot` *Generate a box and whisker plot of membership robustness for all clusters*

---

### Description

This function uses the `lattice` function `bwplot` to generate a box and whisker plot of membership robustness from the result of a call to the `memrob` function.

### Usage

```
membBoxPlot(x)
```

### Arguments

| | |
|---|---|
| `x` | the result of a call to the `memrob` function. |

### Author(s)

Dr. T. Ian Simpson `<ian.simpson@ed.ac.uk>`

## References

Merged consensus clustering to assess and improve class discovery with microarray data. Simpson, T.I., Armstrong, J.D. and Jarman A.P. (submitted).

## See Also

`memroblist-class`,`memrob`

## Examples

```
#load up a test cluscomp result
data('testcmr');

#calculate the membershpi robustness for one of the clustering results
mr <- memrob(testcmr$e1_kmeans_k5);

#plot the bwplot
membBoxPlot(mr);
```

---

`memrob` *Calculate the membership robustness from consensus clustering results*

---

## Description

This function calculates the membership robustness from a `consmatrix` or `mergematrix` class object.

## Usage

```
memrob(x,rm)
```

## Arguments

| | |
|---|---|
| `x` | either a `consmatrix` or `mergematrix` object. |
| `rm` | (optional) if a `mergematrix` object is passed then you must provide a reference clustering structure to calculate cluster robustness against. These structures are stored with every `consmatrix` object in the 'rm' slot. You would normally select a reference matrix for a cluster number matching that of the `mergematrix` (see example below). |

## Value

Returns a list of `memroblist` class objects, one for each cluster, and the full membership robustness matrix as a `memrobmatrix` class object.

## Author(s)

Dr. T. Ian Simpson `<ian.simpson@ed.ac.uk>`

### References

Merged consensus clustering to assess and improve class discovery with microarray data. Simpson, T.I., Armstrong, J.D. and Jarman A.P. (submitted).

### See Also

Also see `cluscomp`, `consmatrix` and `mergematrix`.

### Examples

```
#load cmr (consensus clustering result produced by cluscomp)
data(testcmr);

#calculate the cluster robustness of the consensus matrix for pam where k=4
mr1 <- memrob(testcmr$e1_kmeans_k4);

#show the membership robustness of cluster 1
mr1$cluster1;

#calculate the cluster robustness of the merge matrix in reference to the clustering stru
mr2 <- memrob(testcmr$merge_k4,testcmr$e1_kmeans_k4@rm);

#plot a heatmap of the full membership robustness matrix
heatmap(mr2$resultmatrix@mrm)
```

---

## `memroblist-class` *Class "memroblist"*

---

### Description

Objects of class `'memroblist'` are created to hold the membership robustness scores for the features (e.g. genes) of a cluster.

### Objects from the Class

Objects can be created by calls of the form `new("memroblist", ...)`, although these objects are normally created internally by the `memrob` function.

### Slots

`mrl`: Object of class `"data.frame"` - the membership robustness list itself

### Author(s)

Dr. T. Ian Simpson `<ian.simpson@ed.ac.uk>`

### References

Merged consensus clustering to assess and improve class discovery with microarray data. Simpson, T.I., Armstrong, J.D. and Jarman A.P. (submitted).

### See Also

Also see the `memrob` function/

### Examples

```
showClass("memroblist")

#load a cmr
data(testcmr);

#calculate the membership robustness for agnes, k=4
mr <- memrob(testcmr$e2_agnes_k4);

#get a membership robustness list
mrl <- mr$cluster1;
```

---

## `memrobmatrix-class` *Class "memrobmatrix"*

---

### Description

Objects of class 'memrobmatrix' hold the full membership robustness matrix generated from analysis of a consensus matrix. This includes the calculations of membership robustness for all features (e.g. genes) for each cluster. This can be useful as it allows you to see what conritbution a particular feature (e.g. gene) is making to other clusters. This could resonably be thought of as a measure similar to 'fuzziness' i.e. partial cluster membership. If the value of the membership robustness for a feature is similar in many clusters then that is additional evidence that the feature is not easily placed in any cluster.

### Objects from the Class

Objects can be created by calls of the form `new("memrobmatrix", ...)`, although they are usually generated internally by the `memrob` function.

### Slots

`mrm`: Object of class `"matrix"` - this is the full membership robustness matrix itself and therefore has the same dimensions as the original data object used in the clustering

### Author(s)

Dr. T. Ian Simpson <ian.simpson@ed.ac.uk>

### References

Merged consensus clustering to assess and improve class discovery with microarray data. Simpson, T.I., Armstrong, J.D. and Jarman A.P. (submitted).

### See Also

Also see the `memrob` function.

### Examples

```
showClass("memrobmatrix")

#load cmr
data(testcmr);

#calculate membership robustness
mr <- memrob(testcmr$e1_kmeans_k3)

#get the full membership robustness matrix (matrix itself held in slot 'mrm')
mrm <- mr$resultmatrix@mrm;
```

---

## mergematrix-class *Class "mergematrix"*

---

### Description

Objects of class 'mergematrix' hold the merge matrix in the same way that a consmatrix object holds a consensus matrix. As merge matrices only make sense in the context of the consensus clustering results that were used to generate them we do not store the meta-data for any one consensus clustering parameter set as we do for a 'consmatrix' object. All we need to identify the 'mergematrix' is the cluster number.

### Objects from the Class

Objects can be created by calls of the form `new("mergematrix", ...)`, although they are normally generated by the `cluscomp` function when merge is specfied.

### Slots

- `cm`: Object of class "matrix" - the merge matrix itself
- `k`: Object of class "numeric" - the cluster number (k) value for which the merge was calculated
- `a`: Object of class "character" - always takes the value of 'merge' to identify it as a merge matrix

### Author(s)

Dr. T. Ian Simpson <ian.simpson@ed.ac.uk>

### References

Merged consensus clustering to assess and improve class discovery with microarray data. Simpson, T.I., Armstrong, J.D. and Jarman A.P. (submitted).

### See Also

Also see the `cluscomp` function.

## Examples

```
showClass("mergematrix")

#load the cmr
data(testcmr);

#get a merge matrix object
mm <- testcmr$merge_k4;

#plot a heatmap of the merge matrix
heatmap(mm@cm);
```

---

`wrappers` *Functions to wrap command calls to clustering functions*

---

## Description

These are primarily internal functions called by `cluscomp` to execute clustering runs and are unlikely to be used directly. The wrappers are detailed in the `algorithm.R` file of the `clusterCons` package and the user can add their own wrappers to this to extend the number of algorithms supported. These five wrappers allow the user to specify the conditions under which the corresponding clustering algorithms are run and follow exactly the same specifications as the corresponding `cluster` functions (see `agnes`, `pam`, `hclust`, `diana` and `kmeans`).

## Usage

```
agnes_clmem(x, clnum, params = list())
pam_clmem(x, clnum, params = list())
hclust_clmem(x, clnum, params = list())
diana_clmem(x, clnum, params = list())
kmeans_clmem(x, clnum, params = list())
apcluster_clmem(x,clnum,params = list())
```

## Arguments

| | |
|---|---|
| `x` | A data.frame of numerical values to be clustered which must pass the `data_check` function. This function simply checks that there are no missing values, that all of the data is numeric and that row.names and column.names are unique. This is essential to ensure that individual rows (e.g. genes) and columns (e.g. experimental conditions) can be identified consistently. |
| `clnum` | The number of specified clusters. When using the `cluscomp` function, this will be over-ridden by the cluster range specified using the parameters `clmin` and `clmax` (see `cluscomp` for details). |
| `params` | A list of key, value pairs specifying the parameters to pass to the clustering algorithm. These follow the exact specification of the original functions in the `cluster` package (see `agnes`, `pam`, `hclust`, `diana` and `kmeans`). |

## Value

Returns a data.frame with row.names matching that of the data.

| | |
|---|---|
| `cm` | cluster membership identifier specifying the cluster into which the row has been classified |

### Author(s)

Dr. T. Ian Simpson <ian.simpson@ed.ac.uk>

### References

Merged consensus clustering to assess and improve class discovery with microarray data. Simpson, T.I., Armstrong, J.D. and Jarman A.P. (submitted).

### See Also

cluster, agnes, pam, hclust, diana, kmeans and apclusterK

### Examples

```
#load some data
data(sim_profile);

#run a basic agnes clustering with 3 clusters
cm <- agnes_clmem(sim_profile,3);

#pass some more complex parameters
agnes_params = list(metric='manhattan',method='single');
cm <- agnes_clmem(sim_profile, 3,params=agnes_params);
```

# Index


∗Topic **classes**
auc-class, 5
consmatrix-class, 11
dk-class, 14
memroblist-class, 20
memrobmatrix-class, 21
mergematrix-class, 22

agnes, *23, 24*
agnes_clmem *(wrappers)*, 23
apcluster, *3*
apcluster_clmem *(wrappers)*, 23
apclusterK, *24*
auc, 4
auc-class, *6*
auc-class, 5
aucplot, 6
aucs, *5, 6*, *14, 15*
aucs *(auc)*, 4

bwplot, *18*

checks, 7
clrob, 8, *10*
cluscomp, *4*, *7–9*, 9, *10–13*, *16*, *17*, *19*, *22*, *23*
cluster, *3*, *10*, *23*, *24*
clusterCons *(clusterCons-package)*, 2
clusterCons-package, 2
consmatrix, *8*, *10*, *18*, *19*
consmatrix *(consmatrix-class)*, 11
consmatrix-class, *4–6*, *13*, *15*, *16*
consmatrix-class, 11

data, 12
data_check, *23*
data_check *(checks)*, 7
deltak, 13, *14*, *15*
diana, *23*, *24*
diana_clmem *(wrappers)*, 23
dk-class, *15*
dk-class, 14
dkplot, 15

expressionPlot, 16
expSetProcess, 17

golub *(data)*, 12

hclust, *23*, *24*
hclust_clmem *(wrappers)*, 23

kmeans, *23*, *24*
kmeans_clmem *(wrappers)*, 23

lattice, *3*, *6*, *15*, *16*, *18*

membBoxPlot, 18
memrob, *10*, *18*, 18, *20*, *21*
memroblist, *19*
memroblist *(memroblist-class)*, 20
memroblist-class, *18*
memroblist-class, 20
memrobmatrix, *19*
memrobmatrix *(memrobmatrix-class)*, 21
memrobmatrix-class, 21
mergematrix, *8–10*, *18*, *19*
mergematrix *(mergematrix-class)*, 22
mergematrix-class, *13*
mergematrix-class, 22

pam, *23*, *24*
pam_clmem *(wrappers)*, 23

sim_class *(data)*, 12
sim_profile *(data)*, 12

testcmr *(data)*, 12

validAUCObject *(checks)*, 7
validConsMatrixObject *(checks)*, 7
validDkObject *(checks)*, 7
validMemRobListObject *(checks)*, 7
validMemRobMatrixObject *(checks)*, 7
validMergeMatrixObject *(checks)*, 7

wrappers, 23

xyplot, *6*, *15*, *16*
